# Differential transcript profiling alters regulatory gene expression during the development of *Gossypium arboreum*, *G.stocksii* and somatic hybrids

**DOI:** 10.1038/s41598-017-03431-3

**Published:** 2017-06-09

**Authors:** Liping Ke, Binglun Luo, Liqing Zhang, Mengna Zhang, Xiushuang Yu, Jie Sun, Yuqiang Sun

**Affiliations:** 1 Key Laboratory of Plant Secondary Metabolism and Regulation of Zhejiang Province, College of Life Sciences, Zhejiang Sci-Tech University, Hangzhou, 310016 Zhejiang Province China; 2 College of Life and Environmental Science, Hangzhou Normal University, Hangzhou, 310016 Zhejiang China; 3 The Key Laboratory of Oasis Eco-agriculture, College of Agriculture, Shihezi University, Shihezi, 832000 Xinjiang Province China

## Abstract

Polyploidy or genome doubling (i.e., the presence of two or more diploid parental genome sets within an organism) are very important in higher plants. Of particular interest are the mechanisms in the new microenvironment of the common nucleus, where doubled regulatory networks interact to generate a viable genetic system capable of regulating growth, development and responses to the environment. To determine the effects of whole genome merging and doubling on the global gene expression architecture of a new polyploid, derived from protoplast fusion of the A_1_A_1_ genome of *Gossypium arboreum* and the E_1_E_1_ genome of *Gossypium stocksii*, we monitored gene expression through cDNA-AFLP in the somatic hybrids (*G. arboreum* + *G. stocksii*). The genomic expression patterns of the somatic hybrids revealed that changes in expression levels mainly involved regulatory genes (31.8% of the gene expression profiles), and the AA and EE genomes contributed equally to genome-wide expression in the newly formed AAEE genome from additivity and dominance effects. These results provide a novel perspective on polyploid gene regulation and hint at the underlying genetic basis of allopolyploid adaption in the new microenvironmental nucleus.

## Introduction

The genus *Gossypium* (cotton) currently consists of approximately 45 diploid species that are divided into eight monophyletic groups, each designated by a single letter (“A-G” and “K genome”) and 6 polyploid species^1^. Ancient hybridization between A and D diploids resulted in a new allopolyploid (AD) lineage in the New World approximately 1 million–2 million years ago^2, 3^. Two of the descendant allopolyploid species: *Gossypium hirsutum* (A_1_A_1_D_1_D_1_) and *Gossypium barbadense* (A_2_A_2_D_2_D_2_), as well as two African-Asian A diploids-*Gossypium herbaceum* (A_1_A_1_) and *Gossypium arboreum* (A_2_A_2_), were each independently domesticated for their long, spinnable, epidermal seed trichomes. These four species collectively account for the world’s cotton fiber production, more than 90% of which is provided by upland cotton *G. hirsutum* ^1^. While these polyploid cotton species are currently geographically separated, their monophyletic origin makes the *Gossypium* genus ideal for investigating emergent consequences of polyploidy. Understanding the cotton genome is important for facilitating advances in crop variety development and utilization. Furthermore, the mechanism of polyploid evolution in cotton can be used as an example to understand other polyploid crops^4^.

Polyploidization causes a simultaneous duplication of all nuclear DNA, and some of the genomic consequences of polyploidization could be dramatic^1, 4–7^. In plants, polyploidy is often associated with novel and presumably advantageous ecological attributes, such as range expansion^8^, novel secondary chemistry and morphology^9^, and increased pathogen resistance^10^, although the underlying genetic basis for these novel adaptations remains obscure. The reunion of two diverged genomes in a common nucleus during allopolyploid speciation entails a suite of genomic accommodations^11–13^, including non-additivity of gene expression^14, 15^ and expression partitioning among tissues and organs^16–19^. The most important point for us is the mechanisms by which doubled regulatory networks interact to generate a viable genetic system capable of regulating growth, development, and responses to the environment^20^.

One consequence of polyploidization is the unequal expression of homoeologous loci which was firstly described in cotton^1, 21–24^. Homoeologous gene expression levels were quantified in diploid and tetraploid flower petals of *Gossypium* using the *Gossypium raimondii* genome sequence as a reference. In the polyploid, most homoeologous genes were expressed at equal levels, although a subset had an expression bias for A_T_ and D_T_ copies. The direction of gene expression bias is conserved in natural and recent polyploids of cotton. Conservation of the direction of bias and additional comparisons between diploids and tetraploids suggest that the different regulation mechanisms of gene expression are different^4^. Regardless of the growth stage, tissue, or stress, the degree of bias between duplicated gene pairs is distributed across a spectrum of different expression ratios including the 50:50 ratio of most homoeologous gene pairs^18, 25, 26^.

Another consequence of polyploidization is expression level dominance. Expression level dominance has been characterized by the abundance of a transcript rather than the transcript origin by comparing expression levels in *Gossypium* tetraploids to those in related diploids for a given gene^22^. Expression level dominance of one of the two genomes has been found in leaf^20, 26^ and petal^25, 26^ tissue of interspecific hybrids and natural *Gossypium* polyploids. Expression level dominance has also been observed in other polyploid species such as Coffea^27^, Spartina^6^, and wheat^7^.

Previously, the transcript contributions of the two co-resident cotton genomes have been quantified using custom microarrays^24, 25, 28^, RNA-seq and EST assemblies^26^, and transcriptome RNA-seq^4, 29, 30^. Here, we used the cDNA-AFLP method to measure global genomic expression in the diploid parents (*G. arboreum* and *G. stocksii*) and their somatic hybrids (*G. arboreum* + *G. stocksii*). A modification of the method using complementary DNA (cDNA) samples in the analysis, known as AFLP-cDNA or cDNA-AFLP display, allows the characterization of tissue-specific gene expression patterns^31^ and the detection of gene expression differences in allopolyploids^32–34^. The cDNA-AFLP method is an extremely efficient and sensitive mRNA fingerprinting technique for identifying both common and rare or unknown transcripts^35–37^ and is an open architecture technology for global transcriptional analysis in a non-model plant species^14, 38, 39^. This technique is a robust and high-throughput tool for the analysis of genome-wide gene expression, and it can be used to identify genes that are differentially expressed in allopolyploids. Quantitative cDNA-AFLP was used to monitor variation in the expression levels of cotton fiber transcripts among a population of inter-specific *Gossypium hirsutum* × *G. barbadense* recombinant inbred lines (RILs), proving to be a cost-effective and highly transferable platform for genome-wide and population-wide gene expression profiling^39^. cDNA-AFLP has been used previously in cotton to compare the transcriptomes of two cotton lines (one fertile and the other male sterile)^40^, to identify genes involved in somatic embryogenesis^41^ and to study gene silencing^42^, and to construct genetic maps^39, 43^.

## Results

**Figure 1:**
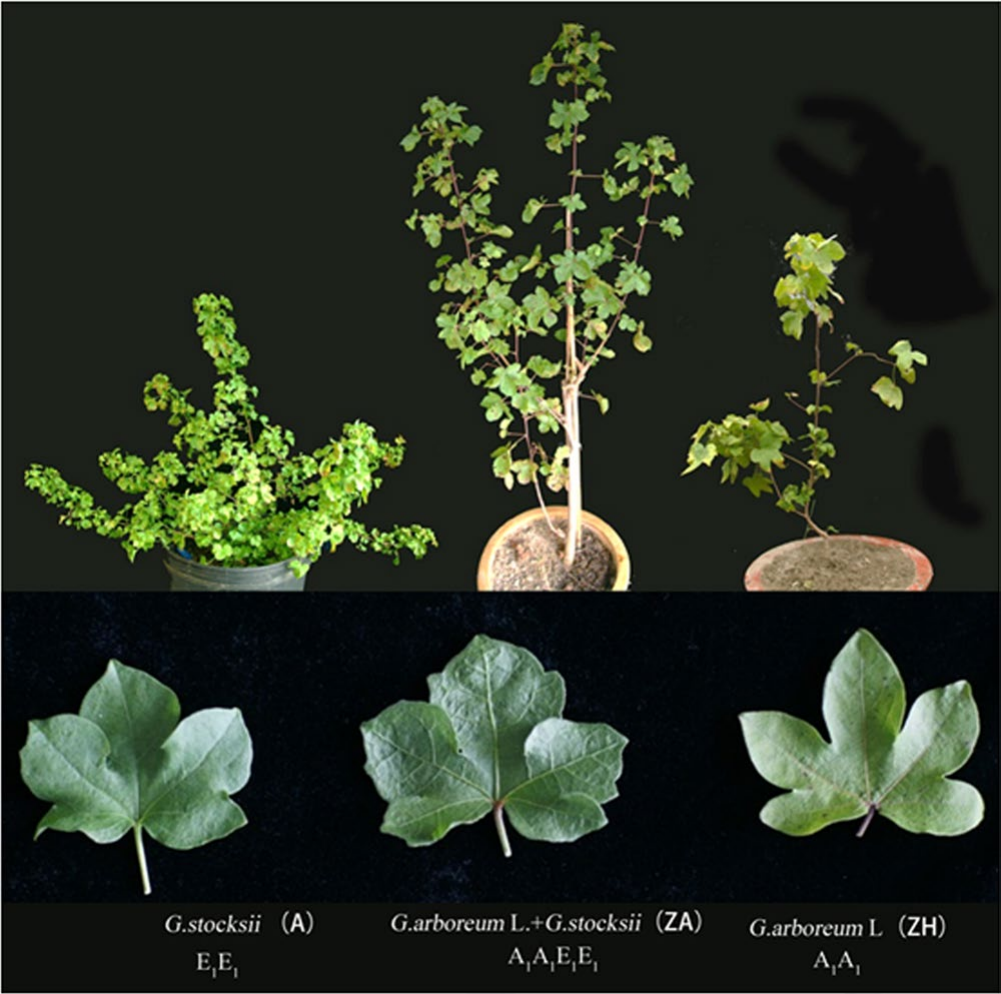
Comparison of the morphology of parental plants and their somatic hybrids.

Differential transcript profiling through cDNA-AFLP was used to investigate and compare transcript changes in the new tetraploid somatic hybrid (*G. arboreum* + *G. stocksii*, a new genotype of A_1_A_1_E_1_E_1_) relative to its diploid parents (*G. arboreum* and wild species *G. stocksii*). The morphology of the somatic hybrid was significantly different to that of the parental plants (Fig. 1A): the leaves of the somatic hybrid were thicker and darker green in color and the plants were more vigorous than the parental plants (Fig. 1B). Leaves at different developmental stages were collected from each of the three species for cDNA-AFLP analysis to investigate the global gene expression changes in the allopolyploid relative to the diploid parental genomes.

### cDNA-AFLP analysis and TDF detection

**Figure 2:**
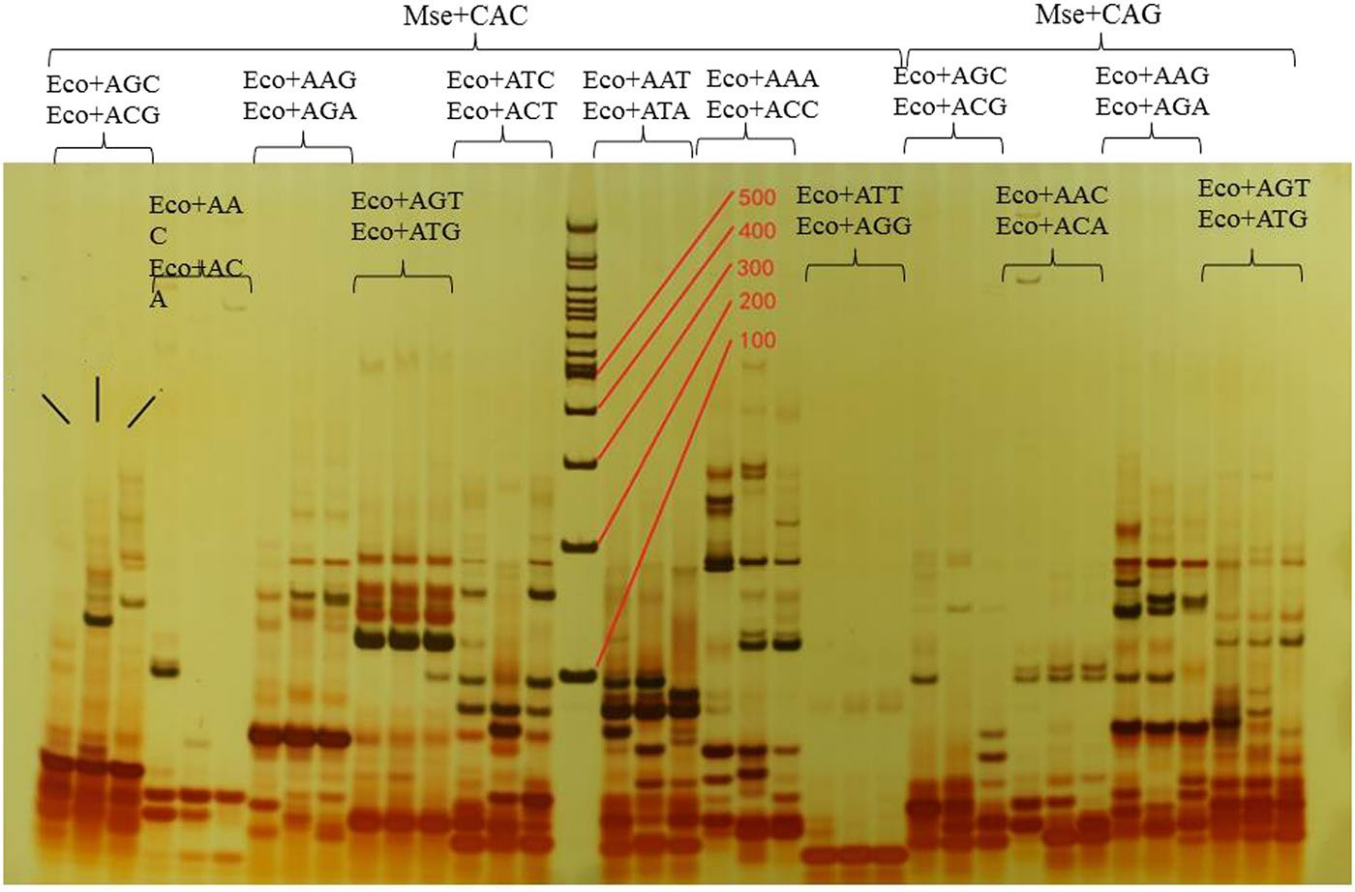
cDNA-AFLP fingerprints of *G. arboreum* (Ga), *G.stocksii* (Gs) and their somatic hybrids (AS). Mse + CAC means Mse I primer plus 3 primer_2 (5′-GAC GAT GAG TCC TGA GTA A – CAC-3′, which are listed in Table 2).

Each of the selective AFLP primer combinations amplified between 100 and 600 fragments, most of which were between 100 and 300 base pairs long. cDNA-AFLP analysis using 64 primer combinations resulted in the identification of more than 6800 clear and unambiguous differentially expressed transcript-derived fragments (TDFs) (Fig. 2).

More than 4000 differentially expressed fragments based on presence/absence or differences in intensity were eluted from the gels, re-amplified and sequenced. The DNA sequence of each TDF was assigned a putative biological function by checking against the cotton EST in the GenBank database (BLASTN/BLASTX). From the sequence alignment, some different TDFs were aligned to the same transcript at different fragments, representing one gene. The sequences of the 1627 sequenced differentially expressed TDFs were aligned. Among the differentially expressed TDFs, the functions of some genes were annotated and not studied in cotton previously. Some of them were cloned with full-length cDNA or genomic DNA sequence and listed in Table 1. TDF113 (Cotton_A_21823) was annotated as Acyl-CoA N-acyltransferase, the same gene was found in the male sterility mutant of cotton described in our previous paper^44^.

**Table 1:** Partial selective TDF functional characterization based on BLASTN and BLASTX algorithms.

TDFs	Locus/gene ID	Annotation	Biological description
TDF211	Cotton_D_gene_10000106	Pectinesterase	cellular organization
TDF280	Cotton_A_09118	Acetylglucosaminyltransferase activity	membrane; Cellular Component
TDF507	Cotton_D_gene_10000255	Disease resistance protein RPS2	defense & response to stimuli
TDF557	Cotton_A_24432 Cotton_A_05650	Nucleobase:cation symporter-1, NCS1	defense & response to stimuli
TDF78	Cotton_A_12744	Family A (phosphoinositide binding specific) member 8	defense & response to stimuli
TDF199	Cotton_A_40094	CCA1 (CIRCADIAN CLOCK ASSOCIATED 1)	defense & response to stimuli
TDF275	Cotton_A_32310	Heat shock protein Hsp20	defense & response to stimuli
TDF57	Cotton_A_21512	RPS2 (RESISTANT TO P. SYRINGAE 2), disease resistance protein RPS2	defense & response to stimuli
TDF116	Cotton_D_gene_10019488	Zeta-carotene desaturase	general and secondary metabolism
TDF167	Cotton_D_gene_10009509	Pyruvate dehydrogenase E2 component (dihydrolipoamide acetyltransferase)	general and secondary metabolism
TDF293	Cotton_D_gene_10003262	Cytochrome P450, E-class, group I	general and secondary metabolism
TDF20	Cotton_A_00679	Auxin responsive SAUR protein	regulation
TDF113	Cotton_A_21823	Acyl-CoA N-acyltransferase	metabolic process; Biological Process
TDF264	Cotton_D_gene_10034692	Serine/threonine protein kinase Cdc7	regulation
	Cotton_A_14578		
TDF1	Cotton_D_gene_10037289	ARF guanyl-nucleotide exchange factor	signal transduction
TDF10	Cotton_D_gene_10001565	Gibberellin receptor GID1	signal transduction
TDF63	Cotton_A_32684	Serine/threonine-protein kinase CTR1	signal transduction
TDF177	Cotton_D_gene_10027381	bZIP transcription factor	Transcriptional regulation
TDF218	Cotton_A_32441	BAK1, brassinosteroid insensitive 1-associated receptor kinase 1	Transcriptional regulation
TDF232	Cotton_A_25131	Arginine/serine-rich splicing factor	Transcriptional regulation
	Cotton_D_gene_10023361		
TDF281	Cotton_A_37559	FAR1-related Transcription factor, Zinc finger	Transcriptional regulation
TDF497	Cotton_D_gene_10033422	MADS-box Transcription factor	Transcriptional regulation
TDF291	Cotton_D_gene_10015764	Translation elongation factor EFG/EF2	translation
	Cotton_A_26279

**Table 2:** Adapters and primers used for cDNA-AFLP analysis.

PRIMER/ADAPTER	5′ sequence 3′
*E*coR I forward adapter	CTC GTA TAC TGC GTA CC
*EcoR* I reverse adapter	AAT TGG TAC GCA GTA
*Mse* I forward adapter	GAC GAT GAG TCC TGA G
*Mse* I reverse adapter	TAC TCA GGA CTC ATC
*EcoR* I + 1 primer	TAC TGC GTA CCA ATT C - **A**
*Mse* I + 1 primer	GAC GAT GAG TCC TGA GTA A - **C**
*Mse* I + 3 primer_1	GAC GAT GAG TCC TGA GTA A - **CAA**
*Mse* I + 3 primer_2	GAC GAT GAG TCC TGA GTA A - **CAC**
*EcoR* I + 3 primer_1	TAC TGC GTA CCA ATT C - **AGC**
*EcoR* I + 3 primer_2	TAC TGC GTA CCA ATT C - **ACG**
*EcoR* I + 3 primer_3	TAC TGC GTA CCA ATT C - **AAC**
*Eco*R I + 3 primer_4	TAC TGC GTA CCA ATT C - **ACA**

**Figure 3:**
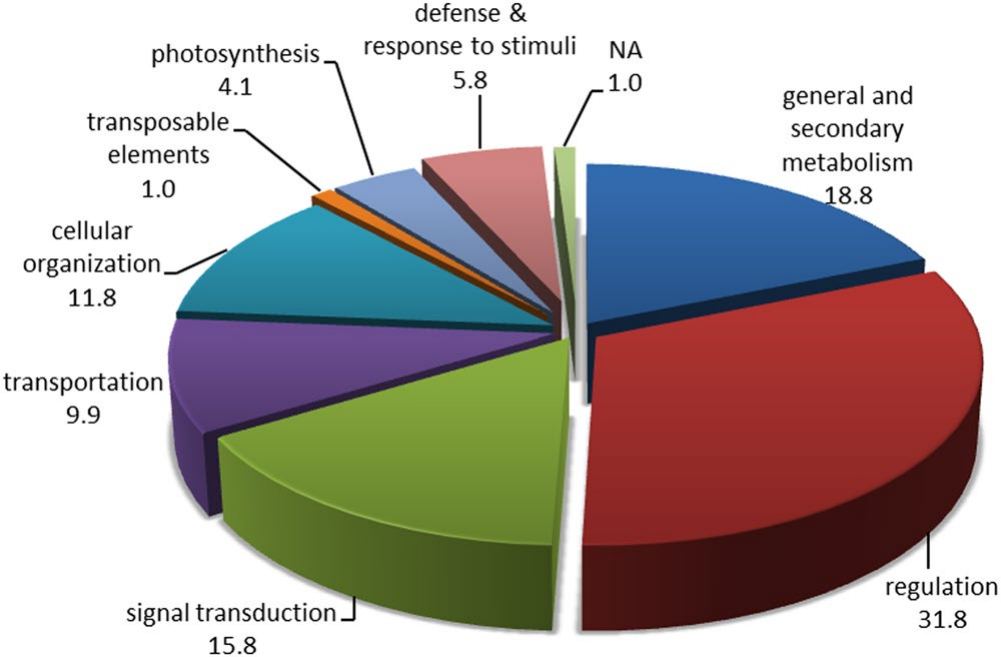
Functional classification of transcript derived fragments: transcript derived fragment categories.

TDFs representing differentially expressed genes were classified into different categories on the basis of their presence/absence (qualitative variation) or differences in expression levels (quantitative variation) among *G. arboreum*, *G stocksii* and their somatic hybrids (Table 3). Different TDFs representing genes controlling biological processes were classified as follows: regulation (31.8%), general and secondary metabolism (18.8%), signal transduction (15.8%), transportation (9.9%), cellular organization (11.8%), defense and response to stimuli (5.8%), photosynthesis & energy (4.1%), transposable elements (1%) and unknown (1%) (Fig. 3).

**Table 3:** Functional classification of transcript derived fragments.

Functional classification	Number	Percentage
General and secondary metabolism	306	18.8
Regulation	517	31.8
Signal transduction	257	15.8
Transportation	161	9.9
Cellular organization	192	11.8
Transposable elements	16	1.0
Photosynthesis & energy	66	4.1
Defense & response to stimuli	95	5.8
NA	17	1.0
Total	1627	100

TDFs representing genes were implicated in the biosynthesis of proteins & amino acids (9%), fatty acids (6%) and carbohydrates (4%) in general and secondary metabolism. TDFs (11.8%) were involved in cellular function and organization, and some TDFs (9.9%) were transporters. Genes involved in signal transduction (15.8%) and regulation (31.8%), including Zn finger binding proteins and ARF guanyl-nucleotide exchange factors, were also detected.

### Differential expression patterns in the two parents and their somatic hybrid

**Figure 4:**
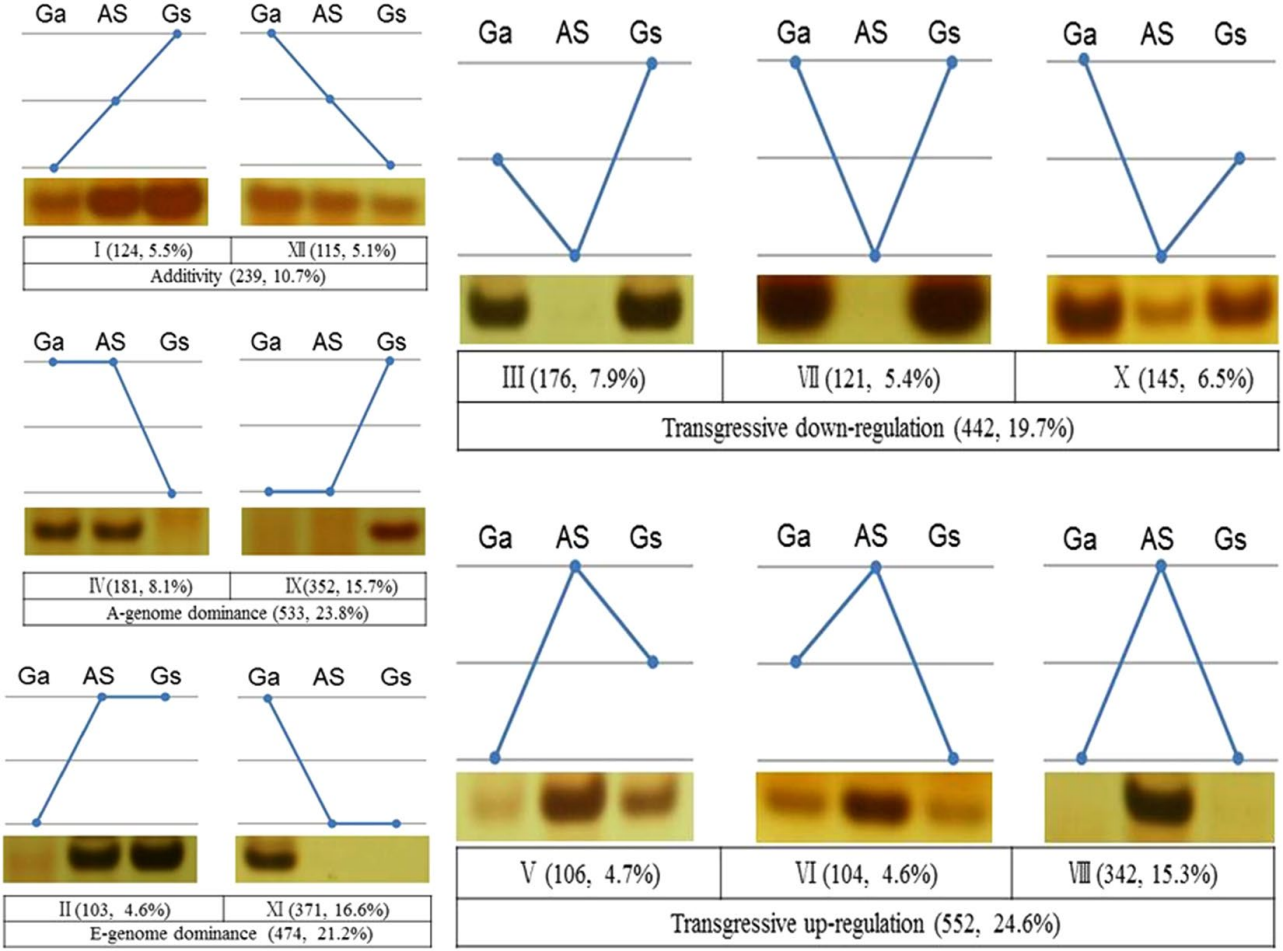
The 12 possible differential expression states in the somatic hybrid relative to its diploid parents. Roman numerals indicate the same categorization as used in Rapp *et al*. (2009), with figures schematizing their respective gene expression pattern for diploid parent *G. arboreum* (Ga: A-genome), tetraploid somatic hybrid (*G.arboreum* + *G.stocksii*: AS, AE genome) and diploid parent *G. stocksii* (Gs: E-genome). Additivity (I and XII), E-expression level dominance (II and XI), A-expression level dominance (IV and IX), transgressive expression lower than either parent (III, VII and X) or transgressive expression higher than either parent (V, VI and VIII).

To detect additivity, transgressive expression, and expression level dominance, we counted 2240 units (one amplification in *G. arboreum*, *G. stocksii* and the somatic hybrid with one combination of primers) of differentially expressed bands for the three individual lines (*G. arboreum*, *G stocksii* and the somatic hybrid) and then grouped genes that showed a change in expression level in the somatic hybrid relative to the expression level in their parents into 12 different categories. These categories were described as additivity (I and XII), E-expression level dominance (II and XI), A-expression level dominance (IV and IX), transgressive expression lower than either parent (III, VII and X), or transgressive expression higher than either parent (V, VI and VIII). The additivity categories (I and XII in Fig. 4) made up 10.7% (239/2240 units), and equivalent expression (approximately 1000 ‘no change’ units were excluded from the total count; these were considered to be mid-parent expression values) of the differentially expressed genes in the somatic hybrid and the two parents, representing the initial stage of the merging genomes, displayed additivity in the allopolyploid somatic hybrid of 5.1% and 5.5% from the AA genome and the EE genome, respectively.

The two effects of dominance (23.8% from the AA genome, 21.2% from the EE genome) and transgressive regulation (19.7% of genes were downregulated, 24.6% were upregulated) contributed to global gene expression in the somatic hybrid at levels of 46% and 44.3%, respectively (Fig. 4).

Approximately 45.2% of genes showed expression level dominance (Fig. 4). Analogous to the expression levels of the two diploid parents and their tetraploid somatic hybrid, the genome-wide expression level dominance resulted from the A genome (23.8%) and the E genome (21.2%), while the direction of expression level dominance showed that gene silencing (15.7% from the A genome; 16.6% from the E genome) occurred simultaneously in the somatic hybrid, and one parent was severe, two – three times for acquired dominant expression (8.1% from the A genome; 4.6% from the E genome) in the somatic hybrid. The degree of biased expression level dominance was the most severe in the somatic hybrid, where the expression levels of 533 genes (23.8% of all genes, categories IV and IX) were statistically equivalent to the A genome parent, compared with 474 genes (21.2%, categories II and XI) for the E genome parent. Thus, gene pairs from the A and E genomes (533 vs 474) exhibited expression level dominance from the A parent and the E parent at the same level.

More genes were transgressively upregulated (24.6%, 552/2240; categories V, VI, and VIII in Fig. 4) than downregulated (19.7%, 442/2240; categories III, VII and X in Fig. 4) in allopolyploids.

**Figure 5:**
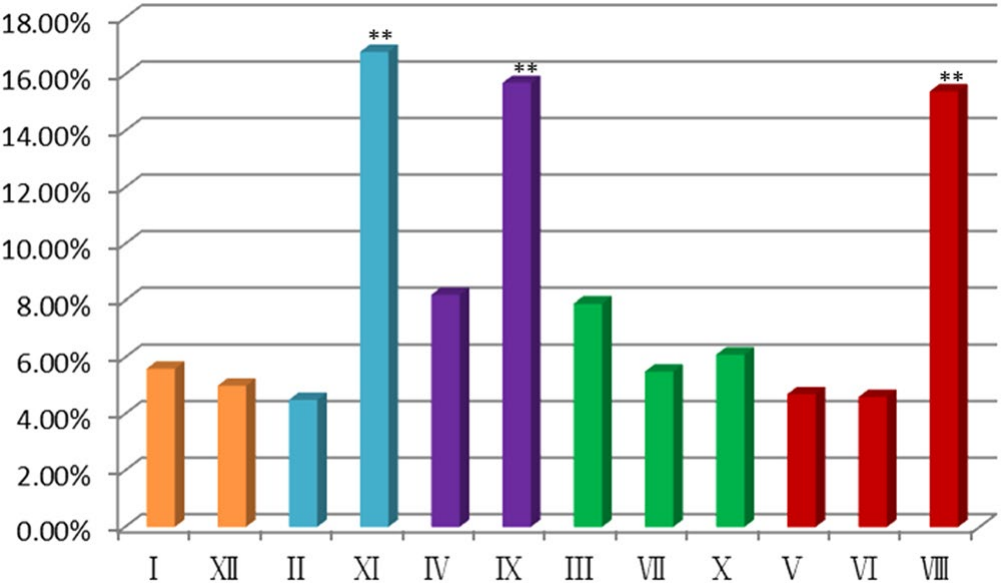
Percentages of differential expression patterns in somatic hybrids and thediploid parents.

Among the transgressively upregulated genes, the percentage of acquired expression in somatic hybrids reached 15.3% (category VIII in Fig. 5), significantly more than the other two types of transgressive upregulation (categories V and VI in Fig. 4, Fig. 5), and significantly more than the transgressive downregulated genes. With regard to the dominance effects of the AA and EE genomes, the proportion of silent expression (category IX for the AA genome and category XI for the EE genome) was significantly greater than dominant expression (category IV for the AA genome and category II for the EE genome) (Fig. 5).

### Relationship between homoeolog-specific expression and expression level dominance

To evaluate how the expression of individual homoeologs relates to joint homoeolog expression, we examined homoeolog expression in each of the 12 categories of differential expression. The results showed that the number of genes showing homoeolog expression bias varied depending on the origin of the parent. In *G. arboreum*, novel expression of the AA genome was 8.1%, and silenced expression was 15.7%; in *G. stocksii*, novel expression of the EE genome was 4.6%, and silenced expression was 16.6%; in the somatic hybrid, novel expression reached 28.6%, and silenced expression was 40.3% from the AA genome and 44.6% from the EE genome (Table 4). For the parental plants, the proportion of silenced expression was the same, whereas the proportion of novel expression in *G. arboreum* was significantly higher than in *G. stocksii*. In the somatic hybrids, the proportion of genes showing novel expression or silenced expression was significantly higher than in the two parental plants. The proportion of homoeologous genes that were silenced depended on the parent of origin in the somatic hybrid.

**Table 4:** Number of genes showing novel expression patterns and putative homoeolog silencing in somatic hybrids.

Taxa	Novel expression (%)	A silencing (%)	E silencing (%)
Ga	8.1%	15.7%	8.1%
Gs	4.6%	4.6%	16.6%
AS	15.3% + 7.9% + 5.4% = 28.6%	15.7% + 4.6% + 4.7% + 15.3% = 40.3%	8.1% + 16.6% + 4.6% + 15.3% = 44.6%

Calculated by dividing the number of genes by 2240 units (each amplification in *G. arboreum*, *G. stocksii* and somatic hybrids with one primer combination).

### Transcript quantification of selected TDFs in five different cotton species

The 12 TDFs were located in the *G. arboreum*, *G. ramondii* or *G. hirsutum* genomes; the CDS sequences derived from the TDFs were obtained, and primers were designed according to the sequences for gene expression analysis in different cotton species, including *G. arboreum* (Ga), *G. stocksii* (Gs), their somatic hybrid (*G. arboreum* + *G. stocksii*, AS), *G. hirsutum* (Gh) and *G. barbadense* (Gb).

The 12 TDFs representing genes for pectinesterase (TDF211), disease resistance protein RPS2 (TDF507), nucleobase:cation symporter-1, NCS1 (TDF557), Heat shock protein Hsp20 (TDF275), Cytochrome P450 (TDF293), Auxin responsive SAUR protein (TDF20), Acyl-CoA N-acyltransferase (TDF113), ARF guanyl-nucleotide exchange factor (TDF1), gibberellin receptor GID1 (TDF10), bZIP transcription factor (TDF177), MADS-box transcription factor (TDF497), RPS2 (RESISTANT TO P. SYRINGAE 2), and disease resistance protein RPS2 (TDF57) (Table 1), showed significantly changed transcript abundance in five different cotton species.

**Figure 6:**
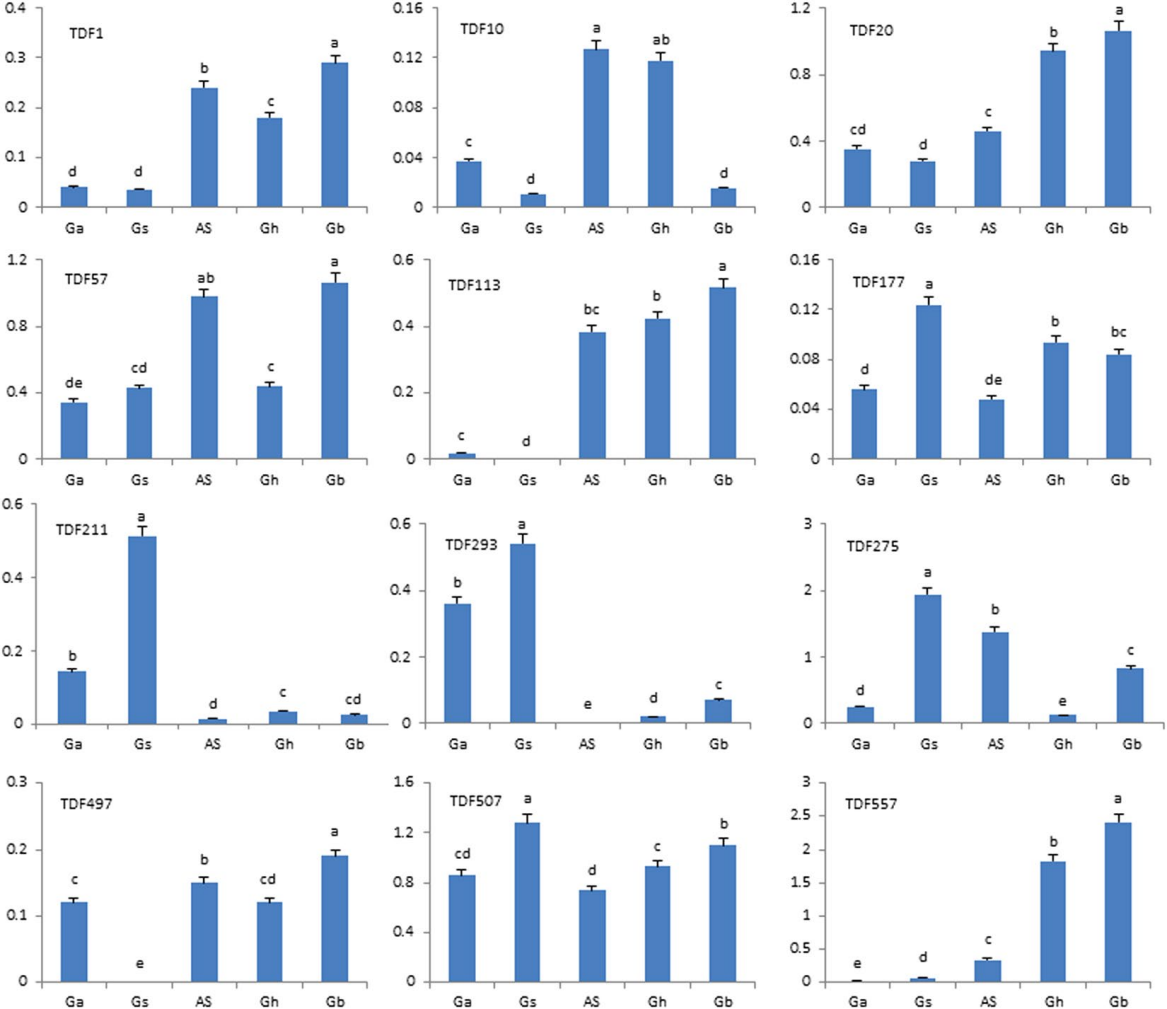
Expression of genes from TDFs in different cotton species. Ga: *Gossypium arboreum*, Gs: *G. stocksii*, AS: somatic hybrid of *G. arboreum* and *G. stocksii*, Gh: *G. hirsutum*, Gb: *G. barbadense*.

A relatively greater abundance of TDFs was observed for TDF557 (nucleobase:cation symporter-1, NCS1), TDF20 (Auxin responsive SAUR protein), TDF113 (Acyl-CoA N-acyltransferase), and TDF1 (ARF guanyl-nucleotide exchange factor) genes in the somatic hybrid, *G. hirsutum* and *G. barbadense*, which are tetraploid cotton species (Fig. 6). For pectinesterase (TDF211) and Cytochrome P450 (TDF293) genes, a relatively lower expression level was observed in all tested tetraploid somatic hybrid cotton species, *G. hirsutum* and *G. barbadense*, significantly greater transcript abundance was observed in diploid wild species of *G. stocksii*, and the two genes were silenced in the somatic hybrid (Fig. 6). Acyl-CoA N-acyltransferase (TDF113) showed acquired expression in the somatic hybrid. NCS1 (TDF557), ARF guanyl-nucleotide exchange factor (TDF1), gibberellin receptor GID1 (TDF10), MADS-box transcription factor (TDF497), and disease resistance protein RPS2 (TDF57) were overexpressed in the somatic hybrid (Fig. 6). In the two parental plants *G. arboreum* and *G. stocksii*, gene silence or very low expression was observed for NCS1 (TDF557), Acyl-CoA N-acyltransferase (TDF113), ARF guanyl-nucleotide exchange factor (TDF1), gibberellin receptor GID1 (TDF10), and MADS-box transcription factor (TDF497) in *G. stocksii* (Fig. 6). MADS-box transcription factor (TDF497) showed significantly higher expression levels in somatic hybrids, *G. barbadense*, *G. arboreum*, and *G. hirsutum*, which have long fibers, and was silent in *G. stocksii*, which only has fuzz fibers (Fig. 6).

The RPS2 gene from TDF507 encoding resistance to *Pseudomonas syringae* protein 2 specifically recognizes the AvrRpt2 type III effector avirulence protein from *Pseudomonas syringae* to guard the plant against pathogens. The RPS2 gene was expressed in all tested cotton species with high levels of expression in wild species *G. stocksii* and *G. barbadense*. Acyl-CoA N-acyltransferase (TDF113) is involved in the metabolism of fatty acids and enters the citric acid cycle, eventually forming several molecules of ATP; it is also involved in the metabolism of carbon sugars as the starting point for the citric acid cycle and in fatty acid metabolism as a balance between carbohydrate metabolism and fat metabolism. Furthermore, acyl-CoA N-acyltransferase is required for the synthesis of flavonoids and related polyketides for elongation of fatty acids. Acyl-CoA N-acyltransferase showed high levels of expression in *G. barbadense* and *G. hirsutum*, and acquired expression in the somatic hybrid. The NCS1gene from TDF557 showed high levels of expression in natural tetraploid cotton of *G. hirsutum* and *G. barbadense*, and in the somatic hybrid of the new synthetic tetraploid cotton.

## Discussion

Here, we report a cotton transcriptome study of a genomic polyploid analyzed by cDNA-AFLP in a somatic hybrid (*G. arboreum* + *G. stocksii*) and two parental plants (*G. arboreum* and *G. stocksii*); our results provide a demonstration of a genomic expression profile after polyploidy in cotton. Changes in gene expression after polyploidy were mainly focused on genes involved in regulation, followed by genes involved in general and secondary metabolism and signal transduction. The contribution effects derived from the parental genomes of AA and EE were equivalent for the double genome of AAEE.

### Changes in gene expression associated with polyploidy

Here, the main changes in gene expression were to genes involved in regulating polyploidy. The differentially expressed genes mainly included genes involved in cell division, the SAUR family, zinc fingers, brassinosteroid insensitive 1-associated receptor kinases, ubiquitin hydrolase and ubiquitin ligase, telomerase activating protein, transcription elongation factors, RNA recognition motifs, DNA-binding motifs, and transcription factors (e.g., MADS-box, TGA, Auxin responsive protein, Zinc finger, bZIP, and K-box). Some differentially expressed genes included seed maturation proteins, pentatricopeptide repeats, tetratricopeptide repeats, and the DDT domain superfamily. These genes are involved in the process of DNA replication, transcription, translation and protein metabolism, and the biosynthesis and interaction of endogenous growth regulators during plant growth and development.

### AA and EE subgenomes have the same contribution to the genome-wide expression of the somatic hybrid

To explore and categorize the expression alterations accompanying polyploid formation, we grouped the differentially expressed genes into the 12 possible patterns of differential expression through clear and unambiguous differentially expressed band patterns. The 2240 units of differentially expressed bands clearly displayed additivity of 5.1% from the A genome and 5.5% from the E genome for genome-wide expression in the newly formed somatic hybrids. The exhibited genome-wide expression level dominance resulted from the A genome (23.8%) and the E genome (21.2%) at the same level. The two effects of additivity and dominance contributed 55.6% to the genome-wide expression of somatic hybrids of two duplicated genomes.

For the two effects, the AA genome contributed 28.9%, and the EE genome contributed 26.7% to the expression of the somatic hybrid genome. The somatic hybrid of the AAEE genome contained two parental genomes of AA and EE, which had the same level of contribution to the new synthetic AAEE genome, different from the bias toward the A genome in a diploid hybrid and natural allopolyploids as described by Yoo *et al*.^26^.

Gene expression patterns in interspecific hybrid F_1_ and leaf transcriptomes from synthetic and natural allopolyploid cotton indicated that genome-wide expression level dominance was biased toward the A genome in the diploid hybrid and natural allopolyploids, whereas the direction was reversed in the synthetic allopolyploid, mainly caused by up- or downregulation of the homoeolog from the ‘non-dominant’ parent^26^.

### Expression effects from the AA and EE genomes

For the dominance effect, the AA and EE genomes almost contributed to genome-wide expression of the AAEE genome at the same level (23% from the AA genome and 21.2% from the EE genome). In the two donor genomes of AA and EE, the silent expression pattern in the dominance effect held the major position in the genome-wide expression of the AAEE genome.

For the transgressive effect in the genome-wide expression of somatic hybrids, the number of genes upregulated (24.6%, 552/2240; categories V, VI and VIII in Fig. 4) was more than those downregulated (19.7%, 442/2240; categories III, VII and X in Fig. 4) in allopolyploids. The result of the polyploidization of the somatic hybrid was an increase in genes transgressively upregulated over those transgressively downregulated (Figs 4, 5). Among the transgressively upregulated genes, acquired expression in somatic hybrids reached 15.3% (categories VIII in Fig. 4), significantly more than the other two types of transgressive upregulation (categories V and VI in Fig. 4). Another remarkable result of polyploidization was an increase of transgressive upregulated genes compared to downregulated genes, especially the activation of the acquired expression of new genes that were silent or not present in both of the parental plants.

For the homoeolog expression in somatic hybrid plants, the proportion of novel acquired expression (15.3%; category VIII in Fig. 4) was roughly equivalent to the proportion of silent expression (13.3%; categories III and VII) while simultaneously expressed in *G. arboreum* and *G. stocksii*. While in the somatic hybrid, the proportion of silenced expression was over 40% from the AA and EE genomes. The phenomena of gene silencing resulting from polyploidization were severe and universal.

In allopolyploid cotton including two natural allopolyploids and an interspecific diploid F_1_ hybrid (*G. arboreum* (A_2_A_2_) × *G. raimondii* (D_5_D_5_)), higher rates of transgressive and novel gene expression patterns as well as homoeolog silencing were observed in natural allopolyploids compared to the F_1_ hybrid or synthetic allopolyploid cotton. Extensive alterations in homoeolog expression bias and expression level dominance accompany the initial merger of two diverged diploid genomes, suggesting a combination of regulatory (*cis* or *trans*) and epigenetic interactions that may arise and propagate through the transcriptome network^26^.

### Validation of TDFs

Because of the large number of differentially expressed gene identified, 12 cloned TDFs were tested by quantitative RT-PCR across the five different cotton species. TDFs with differential expression patterns belonged to genes involved in regulation, general and secondary metabolism, signal transduction, transportation, cellular organization, defense and response to stimuli (Fig. 6, Table 1).

The genes for regulation, general and secondary metabolism, signal transduction, transportation, cellular organization, and defense and response to stimuli were expressed at different levels in the diploid parents and their somatic hybrids; the expression levels of some genes were the same in tetraploid species including the somatic hybrids, *G. hirsutum* and *G. barbadense*, suggesting polyploidization enhanced gene expression; however, some genes became silent after polyploidization. These genes are all candidate sequences for validation of the cDNA-AFLP technique. The identified unigenes could be screened by qRT-PCR for verification of quantitative changes in transcript abundance (gene expression) for the cDNA-AFLP. AFLP based TDFs could represent the identification of differentially expressed genes in genome-wide-expression analysis.

The *RPS2* gene was expressed in all tested cotton species, with high abundance in wild species of *G. stocksii* and *G. barbadense*. The disease resistance (R) protein specifically recognizes the AvrRpt2 type III effector avirulence protein from *Pseudomonas syringae*, interacts with RIN4, and probably triggers plant resistance when RIN4 is degraded by AvrRpt2^45^. In this experiment, *RPS2* had high expression levels in the five cotton species. Serine/Threonine Kinase receptors play a role in the regulation of cell proliferation, programmed cell death (apoptosis), cell differentiation, and embryonic development. The *NCS1* gene (nucleobase:cation symporter-1) showed high levels of expression in the natural tetraploid cotton species *G. hirsutum* and *G. barbadense* and high levels of expression in the somatic hybrid of new synthetic tetraploid cotton, but showed very low expression levels in the diploid parental plants, perhaps related to polyploidization. The expression level changes in these genes occurring in the diploid parental species and their hybrids were validated in the global expression pattern change of the whole genome of the newly synthesized polyploid hybrid.

Genes duplicated by polyploidy (homoeologs) may be differentially expressed in the synthesized hybrid by protoplast fusion of *G. arboreum* and *G. stocksii* compared with their parental species. Compared to previous studies, a surprising level of expression homeostasis was observed in the expression patterns of polyploid genomes; in the new microenvironmental nucleus of somatic hybrids, the main functional classes of changed gene expression were attributed to regulation; the AA and EE genomes showed equal contributions to genome-wide expression of the newly formed AAEE genome from additivity and dominance effects. Mechanisms of gene regulation in the cotton genome warrant further investigation.

## Materials and Methods

The diploid species *G. arboreum* (A_1_A_1_ genome) and the wild species *G. stocksii* (E_1_E_1_ genome) and their somatic hybrids (*G. arboreum* + *G. stocksii*, A_1_A_1_E_1_E_1_ genome) via protoplast fusion were planted in the greenhouse of our campus and used in this experiment. The somatic hybrids of *G. arboreum* + *G. stocksii* were confirmed by cytological examination, molecular markers and ploidy analysis by DNA content with flow cytometry. More than ten plants of each taxon were grown in growth chambers. Plants were grown at 26 °C with a photoperiod of 14 h of light and 10 h of dark and watered as necessary. Samples of fresh young leaves in different developmental stages (germination, seedling, bud, following and boll) were collected (from May to the end of August). Samples were harvested between 9 and 10 AM, immediately frozen in liquid nitrogen and stored at −80 °C before total RNA extraction.

### RNA extraction and cDNA synthesis

Young leaves at different developmental stages were mixed equivalently and ground in liquid nitrogen. Total RNA was isolated from leaves using the RNAprep Pure Plant Kit (Qiagen, GmbH, German) according to the manufacturer’s instructions.

RNA quality was verified on a 1.4% denaturing agarose gel. Total nucleic acids were quantified using a Nanodrop 2000 °C spectrophotometer (Thermo Scientific, USA), and DNA contamination was quantified using a DNA-free Kit (Ambion, USA).

Double-stranded cDNA was synthesized using an iScriptTM cDNA Synthesis Kit (Bio-rad, USA) according to a standard double-stranded cDNA synthesis protocol.

### cDNA-AFLP analysis

A total of 200 ng of double stranded cDNA was subjected to standard AFLP template production according to Vuylsteke *et al*.^46^ with little modification. cDNA was digested with restriction enzymes *Mse*I and *EcoR* I (NEB, England). Digested products were then ligated to adapters with the following sequences: *Mse*I adapter 5′-GACGATGAGTCCTGAG-3′, 3′-TACTCAGGACTCAT-5′; *EcoR* I adaptors 5′-CTCGTATACTGCGTACC-3′, 3′-AATTGGTACGCAGTA-5′. Adapter ligated DNA served as a template for pre-amplification, with PCR parameters of 30 cycles of 30 s at 94 °C, 60 s at 56 °C, and 60 s at 72 °C. The diluted (30-fold) amplified products were used as the template for selective amplification. Equal amounts of pre-amplified products were amplified with primers having selective nucleotides at the 3′ end in a total volume of 20 μl. The primers were listed in Table 2. First selective amplification cycle consisted of 30 s at 94 °C, 30 s at 65 °C, and 60 s at 72 °C; annealing temperature was lowered by 0.7 °C per cycle during the next 12 cycles, followed by 23 cycles at 94 °C for 30 s, 56 °C for 30 s, and 72 °C for 60 s. All PCR reactions were carried out in Applied Biosystem model 9902 Veriti thermal cycler. To each PCR product 7.5 μl of formamide dye (98% formamide, 10 mM EDTA, 0.005% xylene cyanol FF, and 0.005% bromophenol blue) was added, and 7 μl of each sample was loaded onto a pre-warmed 6% polyacrylamide gel using 1x Tris–borate–EDTA (TBE) buffer. Electrophoresis was then run for 2.5 h at 65 W and the gels were silver stained using a silver staining kit (Promega cat. #Q4132, Madison, WI), following the manufacturer’s instructions.

### Transcript-derived fragment (TDF) isolation and re-amplification

Differentially expressed TDFs based on presence, absence or differences in intensity were carefully excised from the gel with a sharp blade to avoid any contaminating fragment(s), eluted in 50 μl of sterile double distilled water, incubated at 95 °C for 15 min and then hydrated overnight at 4 °C. An aliquot of 2 μl was used for re-amplification in a total volume of 25 μl, using the same set of corresponding selective primers and PCR conditions as used for the selective amplification, except that an annealing temperature of 56 °C for 35 cycles was used. PCR products were resolved in a 2% agarose gel; each single band was isolated and eluted using the QIA quick DNA gel extraction kit (Qiagen, USA). The reproducibility of cDNA-AFLP was verified by repeating two times.

### Cloning and sequencing of TDFs

Eluted TDFs were cloned into the plasmid pGEM-T easy® vector (Promega, Madison, USA) and transformed into *E. coli* DH5α following the manufacturer’s protocol and then sequenced. For each TDF, three individual clones were isolated and sequenced. The nucleotide sequences were compared with publicly available cotton EST databases using BLAST sequence alignments. Lists of cloned TDFs, primers and other features are summarized in Table 2.

Sequences of TDFs (with vector sequences trimmed off, where the plasmid was used as the template) were then analyzed for their homology against the publicly available nonredundant genes/ESTs/transcripts in databases of *Gossypium arboreum* L., *Gossypium raimondii* Ulbr., and *Gossypium hirsutum* L. (https://www.cottongen.org/;http://www.ncbi.nlm.nih.gov/BLAST, http://cgp.genomics.org.cn/page/species/blast.jsp, http://www.arabidopsis.org/Blast) using BLASTN and BLASTX algorithms, according to Gupta *et al*.^38^. Then, all the unigenes of 1627 were annotated using a BLASTx search of the UniProt database (http://www.ebi.ac.uk/uniprot/). GO-KEGG-EC annotation was performed based on the Annot8r platform^46, 47^. TDFs were also checked for putative function against the *Arabidopsis* database and the cotton genome database (Institute of Cotton Research of CAAS) using the FASTA tool (http://www.arabidopsis.org/cgi-bin/fasta/nph-TAIRfasta.pl, http://cgp.genomics.org.cn/).

From the aligned and annotated differentially expressed genes, analysis of expression level dominance and homoeolog expression bias, we first explored the data for novel expression (new expression of a gene in a tissue) and homoeolog silencing patterns (no expression of one homoeolog) in the somatic hybrid and parents. Novel expression was inferred when both parental species had no bands for a gene, yet allopolyploids displayed clear bands in all three replicates. If both parental species had clear bands for a homoeolog, but somatic hybrids had no band for the same homoeolog, this was considered silencing. These two cases were eliminated from further analysis, focusing on genes that are expressed in at least one parent and where both homoeologs are expressed in the somatic hybrids. Genes identified as differentially expressed in the somatic hybrid relative to their diploid parents were grouped into 12 possible classes of differential expression (see Fig. 2), that is, expression level dominance, additivity and transgression (outside the range of either parent), according to Rapp *et al*.^20^. Briefly, genes were parsed into these 12 categories (using Roman numerals; see Fig. 2), depending on relative expression levels between the two parents and those of the somatic hybrids. Examined in this manner, genes may display additivity (I and XII), E-expression level dominance (II and XI), A-expression level dominance (IV and IX), transgressive expression lower than either parent (III, VII and X) or transgressive expression higher than either parent (V, VI and VIII).

For each of the 12 categories above (which are based on joint expression levels for both homoeologs), we tabulated homoeolog-specific bands to examine how homoeolog usage for each gene pair was related to total gene expression for each homoeolog pair for each of the 12 categories.

### Quantitative Real Time-PCR

As cDNA-AFLP bands are anonymous, 12 selected TDF fragments were isolated from gels and sequenced to tentatively confirm by qPCR the population-wide quantitative cDNA-AFLP profile. The sequences of these 12 TDFs were aligned against the cotton sequence databases using a Blast algorithm.

The 12 TDFs were subsequently tested by QRT-PCR. Sequence homology of the TDFs with cotton EST sequences allowed the design of gene-specific primers. Expression profiling over the tested cotton species (two natural tetraploid cotton species *G. hirsutum* and *G. barbadense*, somatic hybrids of *G. arboreum* + *G. stocksii*, two parental plants of *G. arboreum* and *G. stocksii*) was carried out using qRT-PCR. For three TDFs, for which the sequence blast result could not discriminate between several possible database accessions, primer pairs were designed for each accession and tested independently by qPCR.

Total RNA was extracted from fresh leaves according to the manufacturer’s instructions (Invitrogen, Carlsbad, CA, USA) and treated extensively with RNase-free DNase I. Double-stranded cDNA was synthesized from 100 ng RNA using iScriptTM cDNA Synthesis Kit (Quanta Quantscript RT kit) according to a standard double-stranded cDNA synthesis protocol. Real-time PCR assays were performed using the SYBR Green Real-Time PCR Master Mix (Promega Gotaq@ qPCR master mix, Madison, USA) and the qRT-PCR reaction was performed using the Eppendorf real-time PCR instrument (Mastercycler ep realplex, Hamburg, Germany). Specificity of the amplified PCR product was determined based on melting curve analysis. Primers for target genes were designed using Premier5 software (Premier Biosoft, Palo Alto, CA). The cotton *Ubiquitin7* gene (*GhUBQ7*, Gen Bank accession number: DQ116441, GhUBQ7F: 5′-GAAGGCATTCCACCTGACCAAC-3′, GhUBQ7R: 5′-CTTGACCTTCTTCTTCTTGTGCTTG-3′) was used as an internal control for the assays. The expression levels of endogenous genes in cotton were obtained and standardized to the constitutive *GhUBQ7* gene expression level. In each study, three independent experiments were conducted. The relative expression were calculated by the 2^−ΔΔCT^ method^48^. Analysis of variance (ANOVA) and means were performed via the statistical software SPSS10.0.

## References

[1] Wendel JF, Cronn RC (2003). Polyploidy and the evolutionary history of cotton. Adv. Agron..

[2] Wendel JF (1989). New world tetraploid cottons contain old world cytoplasm. Proc. Natl.Acad. Sci. USA.

[3] Li FG (2015). Genome sequence of cultivated Upland cotton (*Gossypium hirsutum* TM-1) provides insights into genome evolution. Nat. Biot..

[4] Rambani A, Page JT, Udall JA (2014). Polyploidy and the petal transcriptome of *Gossypium*. BMC Plant Biol..

[5] Salmon A, Flagel L, Ying B, Udall JA, Wendel JF (2010). Homoeologous nonreciprocal recombination in polyploid cotton. New Phytol..

[6] Chelaifa H, Monnier A, Ainouche M (2010). Transcriptomic changes following recent natural hybridization and allopolyploidy in the salt marsh species *Spartina* × *townsendii* and *Spartina anglica* (Poaceae). New Phytol..

[7] Qi B (2012). Global transgenerational gene expression dynamics in two newly synthesized allohexaploid wheat (*Triticum aestivum*) lines. BMC Biol..

[8] Hijmans RJ (2007). Geographical and environmental range expansion through polyploidy in wild potatoes(*Solanum* section *Petota*). Global Ecol.Biogeogr..

[9] Leitch AR, Leitch IJ (2008). Genomic plasticity and the diversity of polyploid plants. Science.

[10] Nuismer SL, Thompson JN (2001). Plant polyploidy and non-uniform effects on insect herbivores. Proc. R. Soc. Lond. B. Biol. Sci..

[11] Osborn T (2003). Understanding mechanisms of novel gene expression in polyploids. Trends Genet..

[12] Adams KL, Wendel JF (2005). Polyploidy and genome evolution in plants. Curr. Opin. Plant Biol..

[13] Chen ZJ (2007). Genetic and epigenetic mechanisms for gene expression and phenotypic variation in plant polyploids. Annu. Rev. Plant Biol..

[14] Wang JL (2006). Genome wide nonadditive gene regulation in *Arabidopsis* allotetraploids. Genetics.

[15] Hegarty MJ (2006). Transcriptome shock after interspecific hybridization in *Senecio* is ameliorated by genome duplication. Curr. Biol..

[16] Adams KL, Liu Z (2007). Expression partitioning between genes duplicated by polyploidy under abiotic stress and during organ development. Curr. Biol..

[17] Adams KL, Wendel JF (2006). Allele-specific, bi-directional silencing of an alcohol dehydrogenase gene in different organs of interspecific cotton hybrids. Genetics.

[18] Hovav R (2008). Partitioned expression of duplicated genes during development of a single cell in a polyploid plant. Proc. Natl. Acad. Sci. USA.

[19] Flagel LE, Udall JA, Nettleton D, Wendel JF (2008). Duplicate gene expression in allopolyploid *Gossypium* reveals two temporally distinct modes of expression evolution. BMC Biol..

[20] Rapp RA, Udall JA, Wendel JF (2009). Genomic expression dominance in allopolyploids. BMC Biol..

[21] Krapovickas A, Seijo G (2008). *Gossypium ekmanianum* (Malvaceae), algodón silvestre de la República Dominicana. Bonplandia.

[22] Grover CE (2012). Homoeolog expression bias and expression level dominance in allopolyploids. Plant J..

[23] Grover CE, Grupp KK, Wanzek RJ, Wendel JF (2012). Assessing the monophyly of polyploid *Gossypium* species. Plant Syst. Evol..

[24] Udall JA (2006). A novel approach for characterizing expression levels of genes duplicated by polyploidy. Genetics.

[25] Flagel LE, Wendel JF (2010). Evolutionary rate variation, genomic dominance and duplicate gene expression evolution during allotetraploid cotton speciation. New Phytol..

[26] Yoo MJ, Szadkowski E, Wendel JF (2013). Homoeolog expression bias and expression level dominance in allopolyploid cotton. Heredity.

[27] Bardil A, De Almeida JD, Combes MC, Lashermes P, Bertrand B (2011). Genomic expression dominance in the natural allopolyploid *Coffea arabica* is massively affected by growth temperature. New Phytol..

[28] Hovav R (2007). A majority of cotton genes are expressed in single-celled fiber. Planta.

[29] Costa V, Angelini C, De Feis. I, Ciccodicola A (2010). Uncovering the complexity of transcriptomes with RNA-Seq. J. Biomed. Biotechnol..

[30] Wang Z, Gerstein M, Snyder M (2009). RNA-Seq: a revolutionary tool for transcriptomics. Nat. Rev. Genet..

[31] Bachem CW (1996). Visualization of differential gene expression using a novel method of RNA fingerprinting based on AFLP: analysis of gene expression during potato tuber development. Plant J..

[32] Comai L (2000). Genetic and epigenetic interactions in allopolyploid plants. Plant Mol. Biol..

[33] Lee HS, Chen ZJ (2001). Protein‐coding genes are epigenetically regulated in Arabidopsis polyploids. Proc. Natl. Acad. Sci. USA.

[34] Madlung A (2002). Remodeling of DNA methylation and phenotypic and transcriptional changes in synthetic *Arabidopsis* allotetraploids. Plant Physiol..

[35] Xiao XH, Li HP, Tang CR (2009). A silver-staining cDNA-AFLP protocol suitable for transcript profiling in the latex of *Hevea brasiliensis* (para rubber tree). Mol Biotechnol..

[36] Fukumura R (2003). A sensitive transcriptome analysis method that can detect unknown transcripts. Nucleic Acids Res..

[37] Song Y (2012). Transcriptional profiling by cDNA-AFLP analysis showed differential transcript abundance in response to water stress in *Populus hopeiensis*. BMC Genomics.

[38] Gupta N, Naik PK, Chauhan RS (2012). Differential transcript profiling through cDNA-AFLP showed complexity of rutin biosynthesis and accumulation in seeds of a nutraceutical food crop (*Fagopyrum* spp.). BMC Genomics.

[39] Claverie M (2012). cDNA-AFLP-based genetical genomics in cotton fibres. Theor. Appl. Genet..

[40] Ma. X (2007). Analysis of differentially expressed genes in genic male sterility cotton (*Gossypium hirsutum* L.) using cDNA-AFLP. J. Genet. Genom..

[41] Leng CX, Li FG, Chen GY, Liu CL (2007). cDNA-AFLP analysis of somatic embryogenesis at early stage in TM-1 (*Gossypium hirsutum* L.). Acta Botanica Boreali-Occidentalia Sinica.

[42] Adams KL, Cronn R, Percifield R, Wendel JF (2003). Genes duplicated by polyploidy show unequal contributions to the transcriptome and organ-specific reciprocal silencing. Proc. Natl. Acad. Sci. USA..

[43] Liu R, Wang B, Guo W, Wang L, Zhang T (2011). Differential gene expression and associated QTL mapping for cotton yield based on a cDNA-AFLP transcriptome map in an immortalized F_2_. Theor. Appl. Genet..

[44] Fu WF (2015). Acyl-CoA N-acyltransferase influences fertility by regulating lipid metabolism and jasmonic acid biogenesis in cotton. Scientific Reports.

[45] Mackey D, Belkhadir Y, Alonso JM, Ecker JR, Dangl JL (2003). *Arabidopsis* RIN4 is a target of the type III virulence effector AvrRpt2 and modulates RPS2-mediated resistance. Cell.

[46] Vuylsteke M, Peleman JD, van Eijk MJ (2007). AFLP-based transcript profiling (cDNA-AFLP) for genome-wide expression analysis. Nature Protocol.

[47] Schmid R, Blaxter ML (2008). annot8r: GO, EC and KEGG annotation of EST datasets. BMC Bioinforma..

[48] Livak KJ, Schmittgen TD (2001). Analysis of relative gene expression data using real-time quantitative PCR and the 2(-Delta Delta C(T)) Method. Methods.

